# Physical Health Monitoring in an Adult Inpatient Mental Health Unit

**DOI:** 10.1192/bjo.2026.11657

**Published:** 2026-06-30

**Authors:** Samina Monir, Tom Barton, Jessica Comley, Scott Ritchie, Ashvanthi Sriranjan

**Affiliations:** Sussex Partnership NHS Foundation Trust, Worthing, United Kingdom

## Abstract

**Aims:**

The aim of this audit is to assess compliance with physical health monitoring for patients admitted in an acute inpatient setting including weight, height, BMI, waist circumference. Also to assess if metabolic risk factors have been identified and whether appropriate risk mitigation measures were taken.

**Methods:**

This is a retrospective audit design where electronic patient records of patients admitted in an acute inpatient unit between November 2024 and August 2025 were reviewed. Information was extracted to determine whether physical health monitoring including weight, height, BMI, waist circumference was completed at baseline (on admission), at 4 weeks, 8 weeks, 12 weeks and subsequently every 3 months. Data was only collected for the duration of the inpatient stay.

Drug charts were reviewed to see whether any changes were made to the treatment regime while inpatient. Discharge summaries were also reviewed specifically the section titled “Plan and Requested Actions” to determine any advice and follow up recommendation were made.

**Results:**

100 patients were admitted in the ward between November 2024 and August 2025. Waist circumference was not measured during the whole length of stay for any patient.

Base line weight was not recorded in 12 patients, height was not recorded in 14 patients. BMI was not calculated for 15 patients.4 patients were not on any psychotropic. Unclear documentation about medication for 3 patients.

37 patients were discharged/transferred before 4 week review.Among the remaining 63 patients, weight was not recorded in 21 (33.33%) patients, height was not recorded in 22 (34.9%) patients. BMI was not calculated in 23 (36.5%) patients. 2 patients didn’t have any psychotropics prescribed.

By 8 weeks, 30 patients were discharged/ transferred. Among the remaining 33 patients,weight was not recorded in 13 (39.39%) patients, height and BMI was not recorded in 14 (42.42%)patients. No psychotropics were prescribed for 1 patient

By 12 weeks, 15 patients were discharged/transferred. Among the remaining 18 patients, weight, height and BMI were not measured in 7 (38.8%) patients.

Before 3 monthly monitoring review point, 13 patients were discharged. Among the remaining 5 patients, weight was not monitored for 1 (20%) patients. 2 (40%) patients didn’t have their height and weight measured.

35 patients had documentation regarding their metabolic risk factor. No patient had clear documentation regarding action taken to mitigate these risks.

**Conclusion:**

This audit shows the need for consistent weight monitoring and implementing a structured traffic-light risk category system to enable early identification and management of antipsychotic-induced weight gain.

